# Traditional physical exercise and depression in older adults: the mediating role of interoceptive awareness and the moderating effect of perceived stress

**DOI:** 10.3389/fpubh.2026.1766902

**Published:** 2026-03-05

**Authors:** Yafei Zheng, Yuanzheng Lin, Qiuhong Zheng

**Affiliations:** 1 Chengdu Sport University, Chengdu, China; 2 Yibin University, Yibin, China; 3 Chongqing College of Mobile Communication, Chongqing, China

**Keywords:** depression, interoceptive awareness, older adults, perceived stress, traditional physical exercise

## Abstract

**Objective:**

This study examined the associations between traditional physical exercise and depressive symptoms among older adults, with particular attention to the mediating role of interoceptive awareness and the moderating effect of perceived stress.

**Methods:**

A cross-sectional survey was conducted with 337 older adults who regularly participated in traditional physical exercise. Measures included the Physical Activity Rating Scale (PARS-3), the Multidimensional Assessment of Interoceptive Awareness (MAIA-2), the Perceived Stress Scale (PSS), and the Patient Health Questionnaire-9 (PHQ-9). Confirmatory factor analysis and model fit indices were examined using AMOS. Mediation and moderation analyses were conducted using SPSS, with bias-corrected bootstrap confidence intervals based on 5,000 resamples.

**Results:**

Traditional physical exercise showed a significant association with lower depressive symptoms. Interoceptive awareness partially explained this association, suggesting that greater awareness of internal bodily states may represent one pathway linking exercise participation and mental health. Perceived stress moderated the relationship between traditional physical exercise and interoceptive awareness, with higher stress levels attenuating this association.

**Conclusion:**

Traditional physical exercise was associated with lower depressive symptoms, and this association was partly accounted for by interoceptive awareness. In addition, perceived stress moderated the strength of the exercise–interoceptive awareness association.

## 1 Introduction

The accelerated aging of the global population and the sustained increase in the burden of chronic diseases have made health promotion and disease prevention an important issue in global public health (1). In this context, the concept of “medical physical integration” is gradually emerging, with its core being the combination of scientific physical exercise and medical practice, thereby playing a comprehensive role in enhancing individual physical fitness, reducing disease risks, promoting rehabilitation, and delaying functional decline (2). In urban contexts, older adults’ mental health is shaped not only by physiological aging but also by socio-demographic conditions and everyday stressors, which may influence both participation in traditional exercise and its psychological benefits (3, 4).

Among various forms of sports, traditional Chinese sports such as Tai Chi and Qigong have high adaptability and safety due to their low impact, gentle movements, and balance of body and mind, making them particularly suitable for middle-aged and older adults as well as chronic disease patients (5). As a culturally embedded form of bodily practice, activities such as Tai Chi and Qigong emphasize slow, mindful movement, ritualized repetition, and collective participation. These features help cultivate a sense of calm, strengthen social connectedness, and foster embodied self-awareness, which have been linked to reduced anxiety, depressive symptoms, and age-related cognitive decline (6, 7).

Despite these well-documented health benefits, the psychological mechanisms underlying the relationship between traditional physical exercise and mental health remain insufficiently understood. In particular, interoceptive awareness—the ability to perceive and interpret internal bodily signals—has been identified as a potential key pathway linking physical activity to emotional well-being (8). Furthermore, contextual factors such as perceived stress may influence this pathway, as high stress can impair bodily awareness and reduce the effectiveness of exercise interventions (9, 10).

Therefore, this study aims to explore how traditional physical exercise influences depression among older adults, with a focus on the mediating role of interoceptive awareness and the moderating role of perceived stress. By clarifying these mechanisms, this research seeks to enrich the theoretical understanding of exercise and mental health, and to provide empirical evidence for developing tailored physical activity interventions that incorporate stress management strategies to optimize psychological outcomes.

## 2 Literature reviews and hypotheses

### 2.1 The direct effect of traditional physical exercise on depression

Traditional physical exercise refers to individuals participating in sports activities with cultural heritage, technical skills, and physical characteristics within a certain time and frequency, such as Tai Chi, martial arts, qigong, etc (11). This type of exercise usually emphasizes the unity of body and mind, respiratory regulation, and coordinated movements. It not only has the function of exercising the body, but also has cultural and psychological value. Depression is a common emotional disorder, characterized by persistent low mood, decreased interest, and loss of pleasure, accompanied by changes in cognitive, behavioral, and physiological functions (12).

Numerous recent systematic reviews and meta-analyses have confirmed that traditional Chinese mind–body exercises such as Tai Chi and Baduanjin are significantly associated with reductions in depressive symptoms among older adults (7, 13). For example, Liu et al.’s (14) systematic review suggests that Tai Chi and Qigong interventions can significantly improve the psychological state of older adults and reduce their depression scores; Park et al.’s (15) systematic review also found that Tai Chi and Qigong can significantly improve the physical function and mental symptoms of patients with schizophrenia or emotional disorders, supporting them as effective non pharmacological interventions. This relationship has been observed in different cultural backgrounds and age groups (16). Therefore, it is speculated that traditional physical exercise is significantly negatively correlated with depression levels.

*H1*: Traditional physical exercise is negatively correlated with depression levels.

### 2.2 The mediating effect of interoceptive awareness

Interoceptive awareness refers to an individual’s ability to perceive, recognize, and interpret internal bodily signals such as breathing, heartbeat, muscle tension, hunger, etc. (17). It not only involves accurate detection of physiological signals, but also includes an individual’s emotional response and self-regulation ability to these signals. Traditional physical exercise such as Tai Chi, Qigong, yoga, etc. often enhances participants’ awareness of internal changes in the body through slow movements and breath control (18), thereby improving their mental health levels.

Previous studies have shown that physical and mental exercises can significantly enhance an individual’s interoceptive awareness (19, 20), while higher interoceptive awareness is negatively correlated with lower depressive symptoms (21). In cross-sectional studies, exercise frequency and intensity were positively correlated with perceived awareness, which partially mediated the relationship between exercise and mental health (8). This indicates that traditional physical exercise reduces depression levels by promoting body awareness, helping to identify and cope with negative emotions. Therefore, a hypothesis is proposed:

*H2*: Interoceptive awareness mediates the relationship between traditional physical exercise and depression levels.

### 2.3 The moderation effect of perceived stress

Perceived stress refers to an individual’s subjective evaluation of the stress caused by environmental events, emphasizing their cognitive and emotional responses to stressful situations (22). It not only depends on the nature of objective events, but is also influenced by individual coping resources, emotional states, and personality traits (23). Previous studies have shown that perceived stress is not only significantly associated with negative mental health outcomes such as depression and anxiety, but also associated with an individual’s level of affective awareness (24, 25). High levels of perceived stress may disrupt the allocation of attentional and regulatory resources toward internal bodily signals, thereby reducing the precision of interoceptive processing and attenuating the interoceptive benefits derived from embodied physical practices (26, 27). Partial cross-sectional studies have found that, at the same level of exercise participation, individuals with high perceived stress have significantly lower levels of perceived improvement in internal sensation compared to those with low stress (8).

Based on the stress vulnerability model, the higher the level of stress, the more likely an individual’s cognitive resources are to be occupied, thereby weakening the effect of exercise intervention on psychological mediating variables such as interoceptive perception (28). Therefore, this study speculates that perceived stress plays a negative regulatory role between traditional physical exercise and internal perception, and the intervention effect on individuals with high perceived stress is weaker.

*H3*: Perceived stress regulates the impact of traditional physical exercise on internal perception, and this effect weakens when perceived stress is high.

### 2.4 Hypotheses and conceptual model

**Figure 1:**
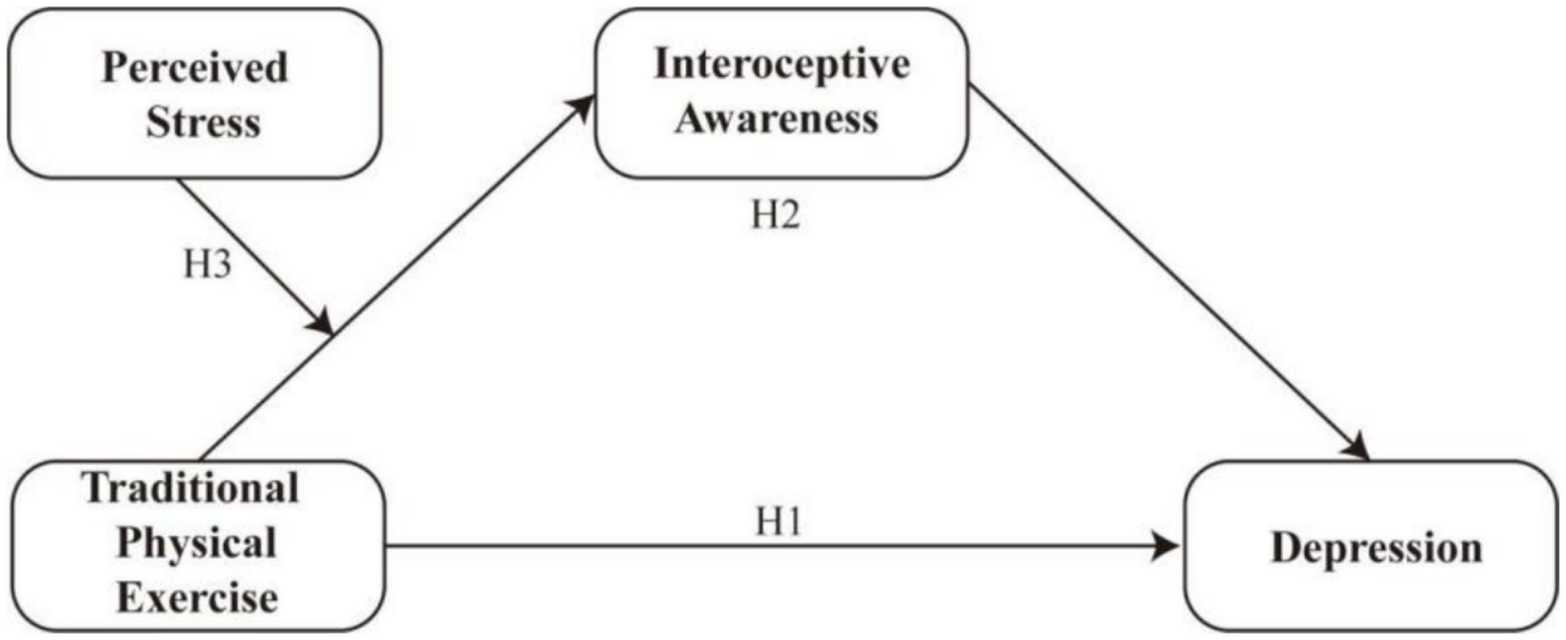
The hypothetical model.

Drawing on the theoretical perspectives outlined above, this study proposes a conceptual framework to investigate the pathways through which traditional physical exercise relates to depressive symptoms in older adults. First, we hypothesize a direct association whereby engagement in traditional physical exercise is linked to lower levels of depression (H1). Second, interoceptive awareness is posited as a mediating mechanism (H2), reflecting the notion that mind–body practices may enhance awareness of internal bodily states, which in turn is associated with better emotional regulation. Third, perceived stress is expected to moderate the relationship between traditional physical exercise and interoceptive awareness (H3), such that the positive association between exercise and bodily awareness becomes weaker under conditions of higher stress. Figure 1 presents the proposed research model and corresponding hypotheses.

## 3 Materials and methods

### 3.1 Participants and procedure

The participants were older adults living in urban communities who had engaged in regular physical exercise at least once per week during the past 6 months and voluntarily agreed to participate in the survey. A combination of convenience sampling and snowball sampling was used to recruit participants from community fitness plazas, park green spaces, and university campuses. The inclusion criteria were as follows: (1) age ≥ 60 years; (2) ability to complete the questionnaire independently; and (3) provision of informed consent. The exclusion criteria included: (1) diagnosed severe cognitive impairment or neurological disorders (e.g., dementia or Parkinson’s disease) that could affect questionnaire completion; (2) acute or chronic illnesses that prevented participation in physical activity; and (3) current use of antidepressant medication or psychiatric treatment that could confound the assessment of depressive symptoms. A total of 337 valid questionnaires were ultimately included in the analysis.

Prior to data collection, participants were informed of the study’s purpose, content, and procedures. Participation was entirely voluntary and anonymous, and all respondents were informed that they could withdraw at any time without consequence. Written informed consent was obtained from each participant. The questionnaire includes measurements of demographic information, participation in traditional sports, perceived feelings, depression levels, and perceived stress. To ensure the reliability and validity of the measurement, a standardized scale widely used internationally was adopted in the study, and the semantic equivalence of the scale was ensured through a translation back translation procedure. This study has been approved by the Research Ethics Committee of Chengdu Sport University, and all procedures comply with the Helsinki Declaration.

### 3.2 Instruments

#### 3.2.1 Traditional physical exercise

Traditional physical exercise was assessed using the Physical Activity Rating Scale (PARS-3) developed by Liang (29). The PARS-3 is a concise and widely applied instrument in Chinese sport and health psychology research for quantifying individuals’ physical activity levels. It evaluates three core dimensions of exercise behavior—intensity, duration, and frequency—each rated on a five-point Likert scale, where higher scores indicate greater engagement in physical activity. The PARS-3 has been extensively used in studies involving middle-aged and older Chinese adults, including research on Tai Chi, Qigong, and other traditional physical activities. Numerous empirical studies have confirmed its good reliability and validity in assessing exercise behavior and its psychological correlates in this population (30, 31).

#### 3.2.2 Interoceptive awareness

Interoceptive awareness refers to an individual’s ability to perceive and interpret internal physiological signals such as breathing, heartbeat, hunger, and tension (32). In the fields of psychology and sports science, interoceptive awareness is regarded as an important mechanism connecting physical activity and mental health, which can help individuals more sensitively perceive emotional and stress changes and regulate behavioral responses accordingly (33). In this study, interoceptive awareness was assessed using the revised version of the Multidimensional Assessment of Interoceptive Awareness (MAIA-2) (20). The MAIA-2 comprises eight dimensions with a total of 37 items, each rated on a six-point Likert scale (0 = never, 5 = always). Several items (e.g., Items 5–10 and 11, 12, 15) are reverse-scored to ensure that higher total scores consistently reflect greater interoceptive awareness. This scale has demonstrated good reliability and validity across diverse cultural contexts and is widely used in sport and health psychology research (34, 35).

#### 3.2.3 Perceived stress

Perceived stress refers to the subjective level of stress experienced by an individual during a specific period of time, reflecting a subjective evaluation of the unpredictability, uncontrollability, and sense of burden in life (22). In sports psychology and health research, perceived stress is widely regarded as an important situational factor that affects psychological processes and behavioral responses, and can regulate the relationship between sports behavior and mental health (36). This study used the Perceived Stress Scale (PSS-10) to measure this variable (37). The PSS-10 contains 10 items rated on a five-point Likert scale (0 = never, 4 = very often), covering the dimensions of perceived tension, lack of control, and self-coping. Items 4, 5, 7, and 8 are reverse-scored to ensure that higher total scores reflect greater levels of perceived stress. Numerous studies have confirmed the good reliability and validity of PSS in different cultural backgrounds and populations, particularly in exploring the relationship between stress and exercise, emotional regulation, and health outcomes (38, 39).

#### 3.2.4 Depression

Depression level is an important indicator for measuring an individual’s negative emotions, cognitive, and behavioral states over a period of time, often used to assess mental health status and its relationship with exercise, stress, and environmental factors (40). This study used the Patient Health Questionnaire-9 (PHQ-9) to measure depression levels. This scale consists of 9 items designed according to the DSM-IV diagnostic criteria for depression, covering symptoms such as low mood, loss of interest, sleep disorders, fatigue, attention difficulties, self-blame and self-injurious thoughts. Participants need to answer on a 4-point Likert scale based on their actual situation in the past 2 weeks (0 = not at all, 3 = nearly every day), with a total score range of 0–27 points. The higher the score, the more severe the depression level. PHQ-9 has been widely used in multiple countries and different populations, with good reliability and validity, especially suitable for application in large-scale health and exercise psychology research (41, 42).

### 3.3 Control variables

To account for potential confounding effects, several demographic and health-related variables were included as statistical controls, following previous research linking these factors to depression, and psychological well-being in older adults. Specifically, the following variables were measured and controlled for in the analysis: age, gender, marital status, education level, household income, and chronic disease status. These factors are known to influence both physical activity engagement and mental health outcomes in later life.

### 3.4 Analysis

Data analyses were conducted using SPSS 20.0, AMOS 24.0, and Hayes’ PROCESS macro (version 3.4).

First, to examine potential common method bias (CMB), Harman’s single-factor test was performed. The first factor accounted for 34.23% of the total variance, below the recommended 40% threshold, suggesting that CMB was not a serious issue in this study.

Second, descriptive statistics, reliability analysis, and correlation analysis were conducted in SPSS to assess the distributional characteristics and internal consistency of the variables. Confirmatory factor analysis (CFA) was then performed in AMOS to evaluate the measurement model’s convergent and discriminant validity as well as overall fit indices (*χ*^2^/df, GFI, CFI, TLI, IFI, SRMR, RMSEA).

Third, the hypothesized mediation and moderation effects were examined using Hayes’ PROCESS macro. Following recommended procedures, all continuous predictors were mean-centered prior to creating interaction terms. The analyses employed heteroskedasticity-robust standard errors (HC3) and included all predefined control variables (i.e., age, gender, marital status, education level, income, and chronic disease status) in each regression equation to ensure model stability.

A bootstrap resampling procedure (5,000 samples) was used to obtain bias-corrected 95% confidence intervals for indirect effects. For significant moderation effects, simple slope analyses were conducted to illustrate the direction and magnitude of interactions under high and low levels (±1 SD) of the moderator.

## 4 Results

### 4.1 Descriptive statistics and correlations among the Main study variables

Table 1 presents the demographic characteristics of the 337 participants. The majority were female, aged between 60 and 70 years, and most were married. In terms of education, the sample was relatively diverse, though junior high school was the most common level attained. The majority reported a monthly income between 1,001 and 3,000 RMB.

**Table 1:** Demographic characteristics of the samples (*N* = 337).

Variable	Category	Frequency (*n*)	Percentage (%)
Gender	Male	151	44.81%
	Female	186	55.19%
Age	60–70	227	67.36%
	71–80	85	25.22%
	>80	25	7.42%
Marital status	Married	309	91.69%
	Divorced/Single	9	2.67%
	Widowed	19	5.64%
Education level	Primary school or below	64	18.99%
	Junior high school	124	36.80%
	High school	84	24.92%
	College or above	65	19.29%
Monthly income (RMB)	<1,000	95	28.19%
	1,001-3,000	191	56.68%
	3,001-5,000	42	12.46%
	>5,000	9	2.67%

Table 2 presents the means, standard deviations, and Pearson correlations among the study variables. The mean scores ranged from 1.20 to 3.03, with standard deviations between 0.53 and 0.86, indicating moderate variability. The variables were moderately correlated in the expected directions. Importantly, no bivariate correlation exceeded the threshold of 0.85, and all VIF values were within acceptable limits, suggesting no concerns regarding multicollinearity.

**Table 2:** Descriptive statistics and correlations among primary variables.

Variable	M	SD	1	2	3	4
1 Traditional physical exercise	1.20	0.86	1			
2 Interoceptive awareness	2.15	0.53	0.402^**^	1		
3 Perceived stress	2.83	0.80	0.050	0.113^*^	1	
4 Depression	3.03	0.75	−0.438^**^	−0.594^**^	−0.014	1

*p < 0.05, **p < 0.01 (two-tailed).

### 4.2 The test of reliability and validity

Table 3 summarizes the reliability and convergent validity of the four latent constructs, evaluated through Cronbach’s *α*, composite reliability (CR), and average variance extracted (AVE). The Cronbach’s α coefficients ranged from 0.836 to 0.949, all exceeding the 0.70 threshold, indicating satisfactory internal consistency. The CR values ranged from 0.850 to 0.949 and the AVE values ranged from 0.570 to 0.676, demonstrating adequate composite reliability and convergent validity. According to Fornell and Larcker (43) and Chin (44), CR values above 0.70 and AVE values greater than 0.50 are considered acceptable, confirming that all constructs in this study possess strong reliability and convergent validity.

**Table 3:** Reliability and convergent validity of the measurement model.

Variable	Cronbach’s α	CR	AVE
Traditional physical exercise	0.836	0.850	0.657
Interoceptive awareness	0.936	0.937	0.651
Perceived stress	0.929	0.929	0.570
Depression	0.949	0.949	0.676

CR, composite reliability; AVE, average variance extracted.

In addition, the overall fit of the measurement model was evaluated using multiple goodness-of-fit indices. As shown in Table 4, the value of *χ*^2^/df was 1.390, which is below the recommended upper limit of 3, indicating an acceptable fit. All incremental fit indices exceeded the commonly accepted threshold of 0.90, including GFI = 0.902, TLI = 0.976, CFI = 0.978, and IFI = 0.978, suggesting that the hypothesized measurement model fits the data well. Moreover, the SRMR value was 0.041, falling below the recommended cutoff of 0.08, and the RMSEA value was 0.034 (90% CI: 0.03–0.04), which is within the range indicative of close model fit. Together, these indicators collectively demonstrate that the measurement model exhibits an excellent overall fit to the data (45).

**Table 4:** Model fit indices for the measurement model.

Fit indices	χ^2^/df	GFI	TLI	CFI	IFI	SRMR	RMSEA [90% CI]
Indices	1.390	0.902	0.976	0.978	0.978	0.041	0.034 [0.03, 0.04]

GFI, goodness-of-fit index; TLI, Tucker-Lewis index; CFI, comparative fit index; IFI, incremental fit index; SRMR, standardized root mean square residual; RMSEA, root mean square error of approximation; CI, confidence interval.

### 4.3 The mediation model analysis

**Figure 2:**
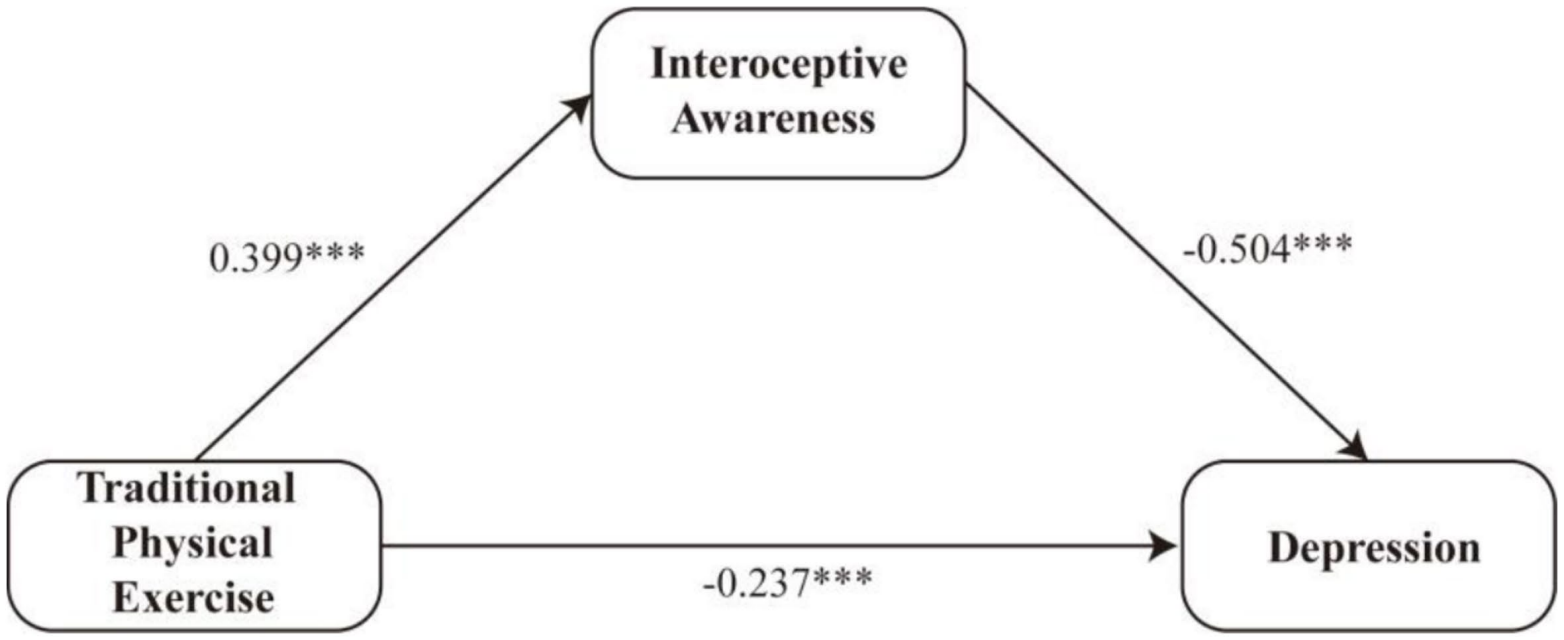
Standardized path coefficients of the mediation model. ***p < 0.001.

Figure 2 illustrates the standardized path coefficients of the proposed mediation model. Traditional physical exercise showed a positive association with interoceptive awareness, which in turn demonstrated a negative association with depression, while traditional physical exercise also exhibited a negative association with depression.

Table 5 presents the regression results for the mediation model. Traditional physical exercise significantly predicted interoceptive awareness (B = 0.245, SE = 0.035, *p* < 0.001). In the second step of the model, both traditional physical exercise (B = −0.208, SE = 0.049, *p* < 0.001) and interoceptive awareness (B = −0.719, SE = 0.058, *p* < 0.001) significantly predicted depression, suggesting that interoceptive awareness may serve as a mediator.

**Table 5:** Regression analysis of the mediation model.

Predictors	Step 1 (interoceptive awareness)			Step 2 (depression)		
	B	SE	*t*	B	SE	*t*
Constant	1.894	0.134	14.12***	5.169	0.192	26.977***
Gender	−0.030	0.054	−0.553	−0.064	0.065	−0.994
Age	−0.025	0.039	−0.646	−0.025	0.052	−0.486
Marital status	0.031	0.058	0.541	0.059	0.063	0.931
Education level	−0.041	0.026	−1.566	−0.068	0.032	−2.118*
Monthly income	0.041	0.037	1.111	−0.092	0.053	−1.722
Traditional physical exercise	0.245	0.035	7.055***	−0.208	0.049	−4.209***
Interoceptive awareness				−0.719	0.058	−12.354***
*R* ^2^	0.173			0.419		
*F*	8.878***			41.069***		

*p < 0.05; ***p < 0.001.

Table 6 further reports the bootstrapped indirect effects. The total effect of traditional physical exercise on depression was significant (B = −0.384, 95% CI [−0.481, −0.287]). The direct effect remained significant (B = −0.208, 95% CI [−0.305, −0.111]), accounting for 54.17% of the total effect. The indirect effect through interoceptive awareness was also significant (B = −0.176, 95% CI [−0.232, −0.127]), explaining 45.83% of the total effect.

**Table 6:** Bootstrapping analysis of the mediation model.

Effect type	Effect	SE	95% CI	Ratio to total effect
Direct effect	−0.208	0.049	[−0.305, −0.111]	54.17%
Indirect effect	−0.176	0.026	[−0.232, −0.127]	45.83%
Total effect	−0.384	0.049	[−0.481, −0.287]	—

Taken together, these findings indicate an associational mediation pattern in which higher levels of traditional physical exercise are linked to lower depressive symptoms both directly and indirectly via interoceptive awareness. These results are consistent with the hypothesized relationships and provide support for H1 and H2.

### 4.4 The moderating model analysis

To examine whether perceived stress moderates the association between traditional physical exercise and interoceptive awareness, PROCESS Model 7 was employed with traditional physical exercise entered as the predictor (X), perceived stress as the moderator (W), and interoceptive awareness as the outcome variable (Y). Gender, age, marital status, education level, and monthly income were included as covariates to adjust for potential confounding influences.

Table 7 presents the regression results of the moderation model. After controlling for demographic variables, traditional physical exercise was positively associated with interoceptive awareness (B = 0.259, SE = 0.038, *p* < 0.001), and perceived stress also showed a positive association with interoceptive awareness (B = 0.066, SE = 0.033, *p* = 0.045). Importantly, the interaction term between traditional physical exercise and perceived stress was statistically significant (B = −0.120, SE = 0.051, *p* = 0.019), indicating that perceived stress moderated the relationship between traditional physical exercise and interoceptive awareness. The 95% bootstrap confidence interval for the interaction effect did not include zero ([−0.221, −0.019]), further supporting the presence of a significant moderation effect, and hypothesis 3 is supported.

**Table 7:** Regression results for the moderation model.

Predictors	Outcome variable: interoceptive awareness			
	*B*	SE	*t*	Bootstrap 95% CI
Constant	2.212	0.130	16.980***	[1.956, 2.468]
Gender	−0.034	0.055	−0.624	[−0.143, 0.074]
Age	−0.028	0.039	−0.714	[−0.105, 0.049]
Marital status	0.019	0.057	0.328	[−0.094, 0.132]
Education level	−0.037	0.025	−1.461	[−0.086, 0.013]
Monthly income	0.036	0.037	0.953	[−0.038,0.110]
Traditional physical exercise	0.259	0.038	6.770***	[0.184, 0.335]
Perceived stress	0.066	0.033	2.011*	[0.002, 0.131]
Traditional physical exercise × perceived stress	−0.120	0.051	−2.354*	[−0.221, −0.019]
*R* ^2^	0.203			
*F*	8.054***			

*p < 0.05; ***p < 0.001; Bootstrap 95% CI based on 5,000 samples.

Simple slopes analyses were conducted to further clarify the nature of the interaction between traditional physical exercise and perceived stress in predicting interoceptive awareness. As shown in Table 8, the association between traditional physical exercise and interoceptive awareness was strongest at low levels of perceived stress (B = 0.355, SE = 0.071, *p* < 0.001). This association remained statistically significant at the mean level of perceived stress (B = 0.259, SE = 0.038, *p* < 0.001). At high levels of perceived stress, the association was still positive but notably weaker (B = 0.163, SE = 0.035, *p* < 0.001).

**Table 8:** Simple slopes of traditional physical exercise predicting interoceptive awareness at different levels of perceived stress.

Perceived stress	Effect	SE	*t*	*p*	95% CI (LL, UL)
Low (−1 SD)	0.355	0.071	5.025	<0.001	[0.216, 0.495]
Mean	0.259	0.038	6.770	<0.001	[0.184, 0.335]
High (+1 SD)	0.163	0.035	4.618	<0.001	[0.094, 0.233]

Bootstrap 95% CI based on 5,000 samples.

**Figure 3:**
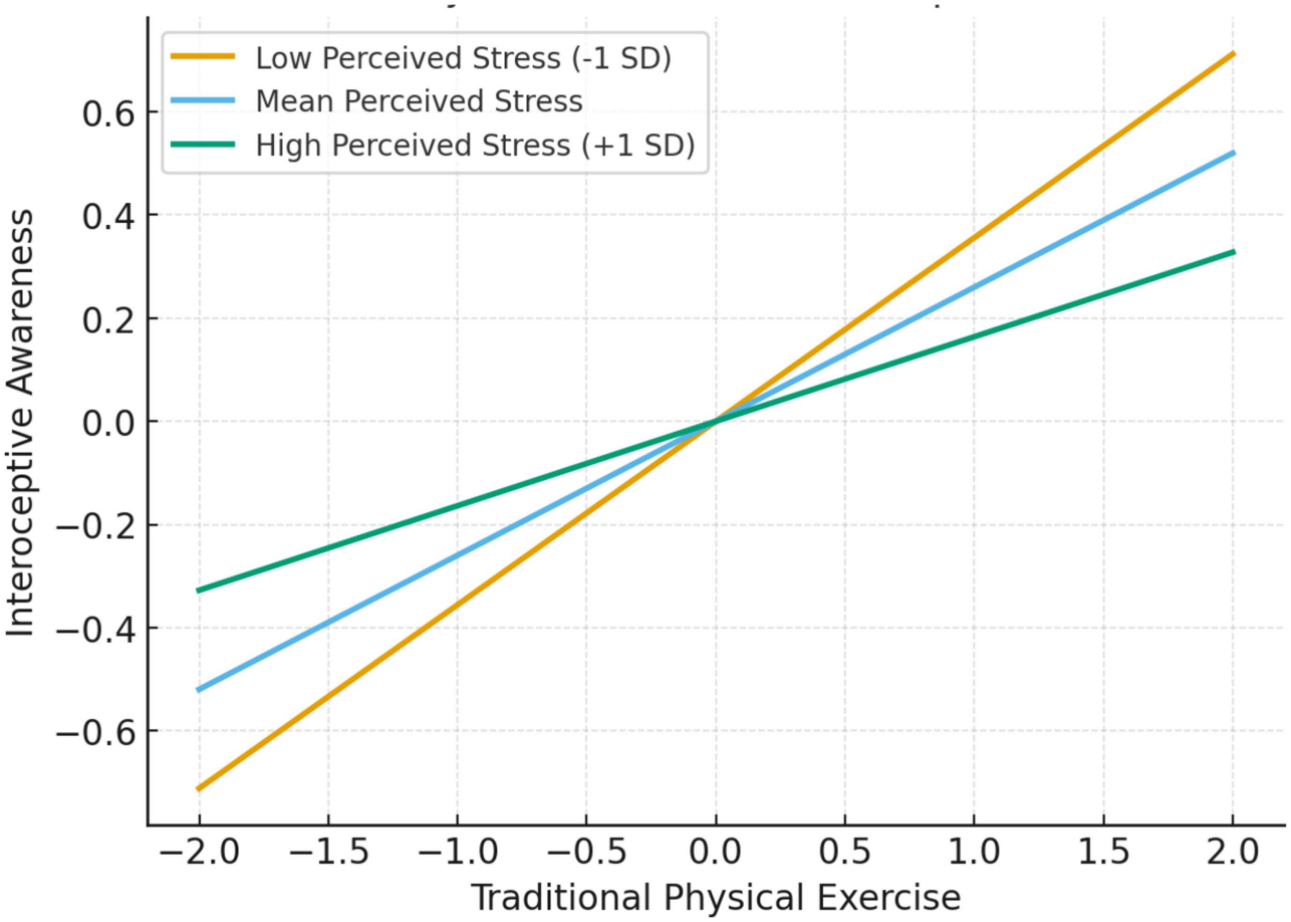
Interaction between traditional physical exercise and interoceptive awareness.

The pattern of these effects is depicted in Figure 3, which illustrates that the slope of traditional physical exercise predicting interoceptive awareness becomes progressively flatter as perceived stress increases. This visual pattern aligns with the statistical results and indicates that perceived stress attenuates the positive association between traditional physical exercise and interoceptive awareness. In other words, traditional physical exercise is more strongly associated with higher interoceptive awareness under conditions of lower perceived stress, whereas this association becomes weaker when perceived stress is elevated. Consistent with these findings, the Johnson–Neyman analysis indicated that the simple slope remained statistically significant when perceived stress was below 1.293 SD.

The Johnson–Neyman analysis showed that the effect remained significant for perceived stress values below 1.293 SD.

## 5 Discussion

### 5.1 The direct effect of traditional physical exercise on depression

The present study found that higher levels of engagement in traditional physical exercise were associated with lower depressive symptoms among older adults, echoing a growing body of evidence on the emotional benefits of movement-based practices (13, 46). Traditional Chinese exercises such as Tai Chi and Qigong differ from conventional aerobic or resistance training in that they integrate slow and continuous movement, controlled breathing, and sustained internal attention. From an embodied cognition perspective, these characteristics may cultivate stable bodily rhythms and a grounded sense of physical presence, which have been linked to emotional balance and psychological well-being in older populations (47, 48).

Beyond their physical components, traditional physical activities also carry notable social and cultural significance. These practices are often conducted in communal spaces, encouraging routine participation, social connection, and shared cultural identity. Prior research suggests that socially and culturally embedded forms of activity may help strengthen interpersonal ties and reinforce a sense of belonging, which is particularly relevant in later life when social networks often shrink (49). In addition, the repetitive, predictable, and ritual-like qualities of practices such as Tai Chi may contribute to feelings of stability and personal agency—psychological constructs that are inversely associated with depressive experiences among older adults (50).

Taken together, the current findings contribute to a broader understanding of traditional Chinese physical exercise as not only a mode of bodily movement but also a culturally grounded, socially supported, and psychologically meaningful activity that aligns with multiple pathways linked to emotional well-being in later life.

### 5.2 The mediating effect of interoceptive awareness

The mediating role of interoceptive awareness provides a meaningful perspective on how traditional physical exercise relates to depressive symptoms among older adults. Practices such as Tai Chi and Qigong emphasize slow movements, coordinated breathing, and sustained bodily attention, which may help cultivate a heightened sensitivity to internal states. This aligns with works suggesting that mind–body activities can function as forms of interoceptive training, supporting clearer recognition and interpretation of bodily signals (27, 51).

Recent research has further demonstrated that higher levels of interoceptive awareness are associated with improved emotional regulation and lower depressive symptomatology (34, 35). Interoceptive processes are increasingly understood as central to adaptive emotional functioning, influencing how individuals interpret physiological cues and respond to stress (27). For older adults, who often experience fluctuating physical sensations and health-related uncertainty, strengthened interoceptive capacities may be particularly relevant to emotional well-being. In this regard, the structured and attentive nature of traditional physical exercise appears to align closely with processes linked to more stable emotional functioning.

Overall, the findings suggest that interoceptive awareness may represent one pathway through which traditional Chinese physical practices are connected to psychological well-being, providing a useful lens for understanding the broader mental health relevance of these culturally embedded forms of movement.

### 5.3 The moderation effect of perceived stress

The moderating effect of perceived stress offers additional insight into how psychological context shapes the association between traditional physical exercise and interoceptive awareness. The observed pattern—stronger associations at lower stress levels and weaker associations under higher stress—aligns with the view that stress can deplete attentional and cognitive resources required for sustained bodily awareness (10). Emerging evidence further indicates that elevated stress may alter the precision and integration of interoceptive signals, thereby constraining individuals’ capacity to effectively process internal bodily information (27).

Traditional practices such as Tai Chi and Qigong involve slow, intentional movements and focused attention on internal sensations. When stress levels are high, individuals may have limited cognitive and emotional capacity to engage fully in these interoceptive processes, as stress-related cognitive load tends to narrow attentional scope and disrupt mindful awareness (52). Conversely, when perceived stress is lower, individuals may be better able to allocate attentional resources toward internal bodily cues, which may enhance the interoceptive benefits of these embodied practices.

Overall, perceived stress appears to influence the extent to which older adults can meaningfully engage with the interoceptive aspects of traditional physical exercise, highlighting the importance of psychological context in understanding these practices.

### 5.4 Practical implication

The findings of this study offer several meaningful practical implications for promoting psychological well-being among older adults. First, the observed associations highlight the potential value of integrating traditional Chinese physical exercise—such as Tai Chi and Qigong—into community-based health promotion programs. These practices are low-cost, accessible, and adaptable for individuals with varying physical capacities, making them well suited for routine implementation in parks, senior activity centers, and community health stations. Encouraging regular participation may help foster structured daily routines, enhance social engagement, and support emotional resilience in later life.

Second, the mediating role of interoceptive awareness suggests that interventions aimed at improving emotional health in older adults may benefit from incorporating elements that cultivate bodily awareness. Traditional physical exercises naturally embed these components through coordinated breathing, mindful movement, and attention to internal sensations. Practitioners and program developers may consider explicitly emphasizing these elements during instruction to enhance their psychological relevance.

Third, the moderating role of perceived stress indicates that individuals experiencing high stress may require additional support to fully engage with the attentional and sensory aspects of these practices. Combining traditional physical exercise with stress-management or psychoeducational components—such as relaxation training or brief mindfulness instruction—may help maximize its emotional benefits.

Collectively, these implications underscore the potential of traditional Chinese physical exercise as a culturally grounded, scalable, and holistic approach for promoting mental health and healthy aging within community settings.

### 5.5 Limitations

Although this study reveals the mechanism by which traditional physical exercise affects depression through interoceptive awareness, and further identifies the moderating effect of perceived stress, several limitations should be acknowledged:

First, this study adopts a cross-sectional design, which only verifies the correlation between variables and cannot make strict causal inferences. Future research could employ longitudinal tracking or randomized controlled trial (RCT) designs to further confirm the causal effects of traditional physical exercise on depression.

Second, this study mainly relies on self-report scales (such as PARS-3, MAIA, PSS, PHQ-9), which may be influenced by social desirability and recall bias. Future studies could incorporate objective physiological indicators such as heart rate variability, cortisol levels, or neuroimaging markers to improve the reliability and validity of the findings.

Third, the sample for this study was drawn from community residents in a specific region, which may limit generalizability due to cultural background, lifestyle habits, and exercise preferences. Future research should expand to diverse regions and cross-cultural populations to enhance the external validity of the results.

Finally, although this study focused on interoceptive awareness and perceived stress, the onset and alleviation of depression may also be influenced by other factors such as social support, lifestyle behaviors, and cognitive biases. Future work could expand the scope of variables to build more comprehensive theoretical models.

## 6 Conclusion

This study highlights the relevance of traditional Chinese physical exercise for the psychological well-being of older adults. Greater participation was associated with lower depressive symptoms, partly through higher interoceptive awareness, and this association was shaped by individuals’ perceived stress levels. These findings underscore the value of traditional mind–body practices as culturally grounded and psychologically meaningful forms of activity in later life. Importantly, these associations should be understood within the broader socio-demographic and urban contexts in which older adults engage in such practices. Continued research using longitudinal and multi-method approaches will help further clarify the mechanisms through which these practices relate to emotional health.

## Data Availability

The original contributions presented in the study are included in the article/Supplementary material, further inquiries can be directed to the corresponding author.
